# Improving attendance to genetic counselling services for gynaecological oncology patients

**DOI:** 10.1186/s40661-018-0059-z

**Published:** 2018-01-10

**Authors:** Hanoon P. Pokharel, Neville F. Hacker, Lesley Andrews

**Affiliations:** 1 Gynaecologic Cancer Centre, Royal Hospital for Women, Sydney, Australia; 2 School of Women’s and Children’s Health, University of New South Wales, Sydney, Australia; 3 Department of Obstetrics and Gynaecology, B P Koirala Institute of Health Sciences, Dharan, Nepal; 4 Hereditary Cancer Clinic, Prince of Wales Hospital, Sydney, Australia; 5 School of Medicine, University of New South Wales, Sydney, Australia

**Keywords:** BRCA1, BRCA2, Genetic testing, Mainstreaming, Hereditary cancer clinic, Efficacy, Effectiveness

## Abstract

**Background:**

Gynaecological cancers may be the sentinel malignancy in women who carry a mutation in BRCA1 or 2, a mis-match repair gene causing Lynch Syndrome or other genes. Despite published guidelines for referral to a genetics service, a substantial number of women do not attend for the recommended genetic assessment. The study aims to determine the outcomes of systematic follow-up of patients diagnosed with ovarian or endometrial cancer from Gynaecologic-oncology multidisciplinary meetings who were deemed appropriate for genetics assessment.

**Methods:**

Women newly diagnosed with gynaecological cancer at the Royal Hospital for Women between 2010 and 2014 (cohort1) and 2015–2016 (cohort 2) who were identified as suitable for genetics assessment were checked against the New South Wales/Australian Capital Territory genetic database. The doctors of non-attenders were contacted regarding suitability for re-referral, and patients who were still suitable for genetics assessment were contacted by mail. Attendance was again checked against the genetics database.

**Results:**

Among 462 patients in cohort 1, flagged for genetic assessment, 167 had not consulted a genetic service at initial audit conducted in 2014. 86 (18.6%) women whose referral was pending clarification of family history and/or immunohistochemistry did not require further genetic assessment. Letters were sent to 40 women. 7 women (1.5%) attended hereditary cancer clinic in the following 6 months.

The audit conducted in 2016 identified 148 patients (cohort 2) appropriate for genetic assessment at diagnosis. 66 (44.6%) had been seen by a genetics service, 51 (34.5%) whose referral was pending additional information did not require further genetic assessment. Letters were sent to 15 women, of whom 9 (6.1%) attended genetics within 6 months.

**Conclusions:**

To improve the effectiveness of guidelines for the genetic referral of women newly diagnosed with ovarian cancer, clinicians need to obtain a thorough family history at diagnosis; arrange for reflex MMR IHC according to guidelines; offer BRCA or panel testing to all women with non-mucinous ovarian cancer prior to discharge and systematically follow up all women referred to genetics at the post-op visit.

## Background

Gynaecological cancers have been recognised as the sentinel cancer in Lynch Syndrome, as well as Hereditary Breast Ovarian Cancer and site specific hereditary ovarian cancer, and provide an opportunity to identify families with mutations in MMR, BRCA1/2 or other gynaecological cancer predisposition genes according to established guidelines [1]. Referral of women newly diagnosed with gynaecological cancer for genetic assessment is based on their personal and family history, their histopathology (high grade serous ovarian cancer in suspected BRCA1/2 mutation carriers or endometrioid, mucinous, clear cell or mixed endometrial or ovarian cancer in suspected Lynch Syndrome) or abnormal mis-match repair immunohistochemistry [2]. As BRCA1/2 mutation carriers can experience meaningful disease control from platinum based chemotherapy and poly ADP-ribose polymerase (PARP) inhibitor treatment upon tumor relapse, there is an increasing clinical need to offer genetic testing to these women [3, 4]. Identification of a BRCA1 or BRCA2 mutation also alerts the woman to her increased risks of breast cancer. Women identified with Lynch Syndrome following a diagnosis of endometrial or ovarian cancer face a lifetime risk of colorectal cancer of around 30%, which is similar to the risk of endometrial cancer for an unaffected woman with a mis-match repair mutation, while the risk of ovarian cancer for an unaffected woman with Lynch Syndrome is around 9%.

With the advent next generation sequencing, panel testing has shown to increase the detection of germline mutations that lead to increased risk of breast, ovarian, and other cancers and can better guide individualized screening measures compared to limited BRCA testing alone. At the same time, multi-gene panel testing is more time-and cost-efficient [5].

Furthermore, predictive testing for family members of women with a pathogenic variant in genes such as the MMR genes, BRCA1/2, RAD51C, RAD51D and BRIP1 provides options for reducing their cancer risk, with screening, surgery or chemoprevention.

**Fig. 1:**
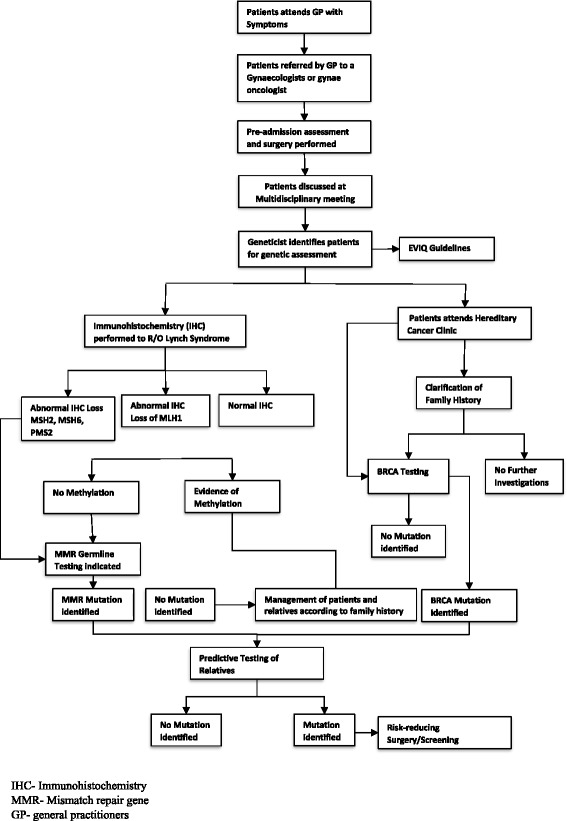
Algorithm for the identification of patients requiring referral for hereditary assessment at the Gynaecologic cancer centre at the Royal Hospital for Women in Sydney

Despite the proven benefits for affected women and their relatives, ensuring all potential cases have genetics assessment is challenging. The normal workflow for genetic referral is shown in fig. 1 [6].

A previous audit of women diagnosed with a gynaecological cancer at the Royal Hospital for Women found that 167 of 462 women who were recommended for further genetics assessment between 2010 and 2014, had not been seen by a genetics service in New South Wales or Australian Capital Territory (NSW/ACT) by February 2016 [7]. This indicates that there is a gap between the efficacy and effectiveness of guidelines to identify gynaecological cancer mutation carriers.

A recent paper described the difference between efficacy and effectiveness with regard to the identification of BRCA1/2 mutation carriers amongst women diagnosed with breast cancer. Efficacy refers to the performance of an intervention under ideal and controlled circumstances. Effectiveness can be defined as the performance of an intervention under “real world” circumstances [8]. It was found that effectiveness of BRCA testing criteria was much lower than efficacy. Hence, the current testing criteria and procedures accompanying BRCA ½ testing are insufficient, and there is room for improving efficacy and effectiveness [8].

Irrespective of family history, 17% of women aged 70 years or younger with newly diagnosed high grade serous ovarian cancer (HGSOC) harbor a germline BRCA1/2 pathologic variant [9]. Despite International guidelines recommending testing for patients with high grade serous ovarian cancer (HGSOC) for germline BRCA1 and BRCA2 pathological variants, the uptake of genetic testing in this patient group remains low, with 19.6% of eligible patients with ovarian cancer declining test [10, 11]. The low rate of genetic testing in eligible patients is likely multifactorial**.** A lack of awareness or misunderstanding of referral guidelines are likely to contribute to non-referral [11].

Additionally the barriers to genetic counselling and testing have been identified in gynaecological oncology patients, including insufficient family history collection, lack of referral, insufficient insurance/cost of the appointment, anxiety for the results, lack of interest, patient/family not wanting to know information regarding cancer risks, and lack of understanding regarding benefits of genetic testing and available preventive measures [12–14].

At the time of our previous audit no formal follow- up recommendation was in place**.** We sought to determine if delayed (more than one year) or short-term (less than one year) follow- up improved adherence to multidisciplinary meeting recommendations regarding genetic assessment and patient attendance at hereditary cancer clinics.

## Methods

All patients undergoing surgery at the Royal Hospital for Women Gynaecologic Cancer Unit are reviewed at the weekly multidisciplinary team (MDT) meeting which is attended by a genetic consultant or a genetic counsellor. Women diagnosed with ovarian, peritoneal, fallopian tube or endometrial cancer warranting further genetic assessment or genetic testing are then referred to the Hereditary Cancer Clinic.

Our previous study identified two cohorts of women. The first cohort included all cases of new or recurrent gynaecological cancer diagnosed between 2010 and 2014 who were recommended for genetics assessment at the weekly multidisciplinary review [7]. The statewide genetics database (Kintrak/Trakgene) identified those who had been assessed by a genetics service in New South Wales or Australian Capital Territory, allowing us to determine those who had not had appropriate genetics follow-up at a public genetics service**.** The second cohort was those women discussed at the review meeting between July 1, 2015 and June 30, 2016 who were recommended but had not had genetics assessment.

The treating gynaecologist of each woman was contacted and advised that genetics assessment was not recorded. They were asked to reply if further information had indicated if further information indicated genetics assessment was not indicated (clarification of family history, MMR IHC), if the woman was deceased, or if genetics assessment had been completed outside of the New South Wales/Australian Capital Territory’s genetics service. Those patients still requiring genetics assessment were sent a letter by the gynaecologist asking them to contact a hereditary cancer clinic from the included list of state-wide services.

Ethics approval was obtained from the Southern Eastern Sydney LDH Human Research Ethics Committee. HREC ref. no: 14/170(LNR/15/POWH/229).

## Results

**Fig. 2:**
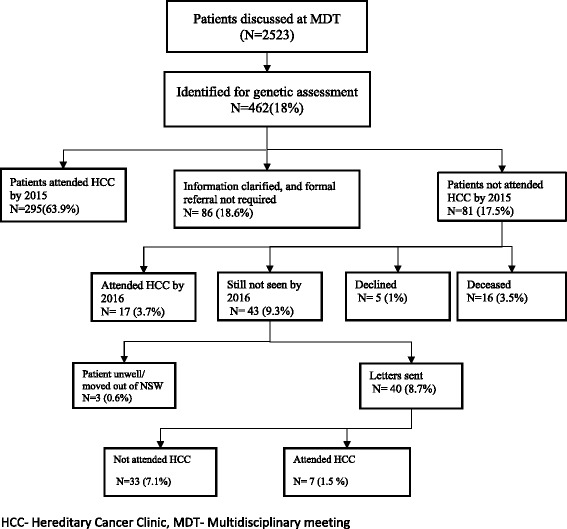
Outcome of case review from 2010 to 2014 (Cohort 1)

Of the 462 patients in cohort 1, 295 (63.9%) attended the Hereditary Cancer Clinic and 86 (18.6%) were deemed by a genetic counsellor to not require formal referral following clarification of family history or mismatch repair gene immunohistochemistry (MMR IHC) status. This left 81 patients (17.5%) who had not attended as recommended. 17 (3.7%) of these consulted a hereditary cancer service between initial ascertainment and this review. Review of the medical records of the remaining 64 patients (13.9%) indicated that 16 (3.5%) were deceased, and 5 (1%) had declined a genetics appointment leaving 43 patients (9.3%) suitable for genetics assessment who had never been seen by a Hereditary Cancer Clinic service. The treating gynaecologic oncologist was contacted; 2 (0.4%) were considered too unwell to attend and 1 (0.2%) had moved out of New South Wales (Fig. 2). Formal letters were sent to 40 patients (8.7%). After 6 months follow up, there were still 33 patients (7.1%) who failed to present to a Hereditary Cancer Clinic in New South Wales or Australian Capital Territory.

**Fig. 3:**
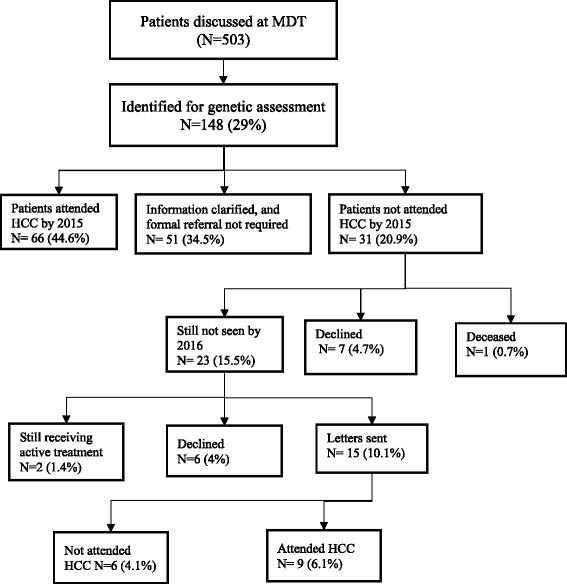
Outcome of case review from 2015 to 2016 (Cohort 2)

The audit of the 503 gynaecological cancer cases discussed at the multidisciplinary meetings in the year July 1 2015–June 30 2016 (cohort 2) identified 148 (29%) who were appropriate for genetics assessment. Of these, 66 (44.6%) had been seen by a genetics service by March 1, 2017, and 51 (34.5%) did not require further assessment after clarification of family history or immunohistochemistry status. Thirty-one patients (20.9%) had not attended a Hereditary Cancer Clinic by March 1, 2017. Of these, 7 (4.7%) declined genetic referral, and 1 (0.7%) was deceased. Treating gynaecological oncologists were contacted regarding the remaining 23 women (15.5%). They reported 6 patients (4%) had declined genetic assessment and 2 patients (1.4%), who were currently undergoing active treatment, intended to attend a hereditary cancer clinic after completion of their treatment. Fifteen patients (10.1%) were sent letters. After 6 months follow-up, 6 patients (4.1%) had still not attended the hereditary cancer clinic (Fig. 3).

## Discussion

Our audits of two retrospective cohorts of women diagnosed with gynaecological cancer has shown that despite having a member of a hereditary cancer team at multidisciplinary review meetings to identify women needing further assessment, approximately 10–15% of women do not receive that assessment. Further, we have demonstrated that information essential for determination of suitability for genetics assessment is frequently not available at the weekly meeting, such as detailed family history or MMR IHC status.

In an effort to optimise uptake, we have trialled a joint initiative between the gynaecological team and the hereditary cancer service to identify and contact non-attenders. Follow up of our original cohort with a letter indicated that late follow up improved attendance by only 1.5% (7/462). Short term follow- up of the second cohort (diagnosed 2015–2016) improved attendance by 6.1% (9/148). Hence, we recommend following up with patients in the short term, rather than the long term.

This process would optimise genetic assessment of gynaecological oncology patients, but requires ongoing interaction between both oncology and genetics services.

As an alternative to referral to a genetic service for women diagnosed with ovarian cancer, the introduction of “mainstreaming,” whereby genetic testing of BRCA1/ 2 or panel of genes is done by the treating gynaecological or medical oncologist at diagnosis of primary or recurrent disease, has the potential to improve appropriate genetic assessment [15]. As a hybrid of mainstreaming, a genetic counsellor with specialized training and experience in familial cancer genetics, directly deployed into a gynaecologic oncology outpatient clinic and during chemotherapy sessions [15] has been reported to improve the uptake rate.

Our audit also identified that 18.6% and 34.5% patients in cohort 1 and 2 respectively who were identified as needing clarification of family history or immunohistochemistry when discussed at the meeting less than two weeks after surgery, were later found not to have indications for further genetic assessment. In most cases this was because family history information was incomplete or inaccurate, especially with confusion between ovarian, uterine and cervical cancers. With time, many women can gather information which is not available at the time of surgery (Figs. 2 and 3). Family history has been the foundation for genetic assessment and the basis for identifying patients at increased risk. Even when not providing full genetic assessment and testing services, oncologists are in a position to identify patients who may be at increased risk of cancer by recognizing the signs of an inherited syndrome. The general recommendation is to get a three-generational family history from all patients [16, 17]. Ideally, this history should include information on first-, second- and third- degree relatives, including the type of each primary cancer, age at diagnosis, age at death, cause of death, and environmental exposures of all relatives with cancer. Many patients may not know these details on all family members at their first visit, so it is important to regularly update the history [18]^.^ This is particularly important for the gynaecological oncologist, as ovarian and endometrial cancers are the sentinel cancer for many women with either a BRCA1 or 2 mutations or Lynch Syndrome, and can be facilitated by a family history questionnaire.

In a number of cases, routine MMR immunohistochemistry had not been done, prompting further assessment, however this could be accomplished if it was integral to histopathological examination for all endometrial cancer, with or without an upper age limit of age of 60 [19], and was included in the request by the gynaecological surgeon (Table 1).

**Table 1:** Four steps to appropriate genetic assessment in gynaecological oncology for the first routine oncology follow-up visit (3-6 months)

1. Non-mucinous ovarian, fallopian tube or primary peritoneal cancer		BRCA testing alone or included in a panel
2. Histopathology	Mucinous ovarian cancer	MMR IHC
	Endometrioid or clear cell ovarian cancer	MMR IHC
	Endometrial cancer	MMR IHC
3. Family History	Ovarian cancer <50	Refer to hereditary cancer clinic for further assessment
	Breast cancer <50	Refer to hereditary cancer clinic for further assessment
	Endometrial or GIT cancer	MMR IHC
4. Post-operative follow up visit	Are investigations completed? Has patient attended hereditary cancer clinic if referred?	

By the time the patient is discharged, the gynaecological oncologist should have clear indications for the genetic management of the patient. This is particularly important for tertiary referral centres, because the patient may live remotely and have difficulty accessing a local genetic service (Table 1).

Our study shows that 16 patients (3.5%) in cohort 1 had deceased at the time of writing, which emphasizes the importance of early follow-up and intervention. In our opinion, the best way not to lose the patients recommended from the multidisciplinary meeting would be for patients to consult oncologists and the genetic counsellors on the same day at the post-operative visit.

A model of including a genetic counsellor in gynaecological cancer care [16, 20] has been shown to enable accurate family history assessment and appropriate IHC to be done, as well as concurrent genetic testing where indicated. This avoids patients being lost to follow up or becoming too ill or passing away before genetics assessment can be completed. Embedding a genetic counsellor in the cancer clinic proved effective, increasing uptake of genetic testing in eligible patients to over 90%.The median time from referral to delivery of genetic testing results was less than five months [16].

## Conclusion

We have demonstrated in our recent cohort that 91.7% of eligible women had received genetic assessment, which seems effective and shows the small gap between the efficacy and effectiveness of guidelines for women diagnosed with gynaecological cancer. Even though, family history is often not helpful in determining genetic risk, effort taking a detail family history will not harm in selecting high risk patients for genetic testing. It is important to note that almost half of patients with a BRCA1/2 mutation and ovarian cancer have no family history of breast or ovarian cancer. For women with high-grade serous tubo-ovarian carcinomas, should be referred for genetic testing irrespective of their family history. A brief 4 step (Table 1) process for gynaecological oncologists is proposed to improve the effectiveness of guidelines for the genetic assessment of women with gynaecological cancer.

While mainstreaming is being adopted by some treating specialists, there will remain a cohort of women for whom hereditary cancer clinic referral is indicated, such as those with gynaecological cancers other than serous histology who have indications for genetic assessment.

Additionally, this model of improving the effectiveness of referral guidelines can be used for patients identified at other multidisciplinary meetings e.g.-breast or colorectal.
